# Metagenomic Analysis Reveals Three Novel and Prevalent Mosquito Viruses from a Single Pool of *Aedes vexans nipponii* Collected in the Republic of Korea

**DOI:** 10.3390/v11030222

**Published:** 2019-03-05

**Authors:** Mark A. Sanborn, Terry A. Klein, Heung-Chul Kim, Christian K. Fung, Katherine L. Figueroa, Yu Yang, Edward A. Asafo-adjei, Richard G. Jarman, Jun Hang

**Affiliations:** 1 Viral Diseases Branch, Walter Reed Army Institute of Research, Silver Spring, MD 20910, USA; mark.a.sanborn9.ctr@mail.mil (M.A.S.); christian.k.fung.ctr@mail.mil (C.K.F.); katherine.l.figueroa.ctr@mail.mil (K.L.F.); yu.yang2.ctr@mail.mil (Y.Y.); richard.g.jarman.mil@mail.mil (R.G.J.); 2 Force Health Protection and Preventive Medicine, MEDDAC-Korea/65th Medical Brigade, Unit 15281, APO AP 96271-5281, USA; terry.a.klein2.civ@mail.mil (T.A.K.); hungchol.kim2.ln@mail.mil (H.-C.K.); 3 Pathology Branch, Walter Reed Army Institute of Research, Silver Spring, MD 20910, USA; edward.a.asafo-adjei.civ@mail.mil

**Keywords:** emerging viruses, metagenomics, arbovirus, mosquitoes, Aedes, bunyavirus, picornavirus, sobemovirus

## Abstract

Arboviruses continue to be a significant global health concern. The unbiased metagenomic analyses of mosquito-borne and mosquito-specific viruses are useful to understand viral diversity and for the surveillance of pathogens of medical and veterinary importance. Metagenomic analysis was conducted on 6368 mosquitoes (736 pools), covering 16 species from 18 locations throughout the Republic of Korea (ROK) in 2016. In this report, we describe three viruses detected in a single pool of *Aedes vexans nipponii* collected at Yongsan U.S. Army Garrison, located in a densely populated district of Seoul, the ROK. The three novel viruses, designated as Yongsan bunyavirus 1 (YBV1), Yongsan picorna-like virus 3 (YPLV3) and Yongsan sobemo-like virus 1 (YSLV1), share sequence and structural characteristics with members belonging to the family *Bunyaviridae*, order *Picornavirales*, and family *Solemoviridae*, with shared RNA-dependent RNA polymerase (RdRp) amino acid identities of 40%, 42% and 86%, respectively. The real-time reverse transcription and polymerase chain reaction (RT-PCR) of 3493 *Aedes vexans nipponii* (257 pools) showed a high prevalence of YBV1 and YSLV1 viruses, which were present in 65% and 62% of tested pools, respectively. This study highlighted the utility of a metagenomic sequencing approach for arbovirus discovery and for a better understanding of the virome of potential medically relevant vectors.

## 1. Introduction

Mosquitoes have a worldwide distribution and vector species have been spreading with increasing speed in recent years due to environment and climate changes, massive global trade and international travel (http://vectormap.si.edu/index.htm) [1]. A number of mosquito species can vector pathogenic microorganisms that are of medical and veterinary importance [2,3]. Mosquito-borne pathogens are responsible for a variety of significant diseases, such as malaria, yellow fever, dengue fever, Zika fever, chikungunya, Japanese encephalitis and many others [3,4,5]. These geographically endemic diseases are responsible for enormous economic burdens and severe disturbances in both developed and underdeveloped regions, communities and patients and their families [2,6,7,8,9,10]. The unbiased metagenomic analysis of field-captured mosquitoes using advanced next-generation sequencing (NGS) technology has greatly facilitated the surveillance of known pathogens and has accelerated the discovery of novel agents, e.g., viruses, bacteria (e.g., *Rickettsia* and *Borrelia* spp.), protozoa (e.g., malaria) and fungi, that are associated with mosquitoes and are known and potential human or animal pathogens [11,12,13,14,15,16,17]. Further metagenomics analyses are necessary to better understand the diversity and abundance of mosquito-borne pathogens and novel agents to better assess the epidemiology of emerging and reemerging mosquito-borne pathogens [18].

Several viruses and rickettsia in mosquitoes of different species in the Republic of Korea (ROK) have previously been identified using traditional culture isolation, PCR screening and metagenomic analysis [11,19,20,21,22]. In our recent large metagenomic study on mosquitoes collected from multiple locations in the ROK in 2016, three novel virus genomes were identified in one pool of 16 *Aedes vexans nipponii*, which is one of the most abundant mosquito species in the ROK. The subsequent analysis of additional *Ae. vexans nipponii* pools resulted in a high rate of positive pools for bunya-like and sobemo-like viral nucleic acids.

## 2. Materials and Methods

### 2.1. Mosquito Collections

Adult mosquitoes were collected using New Jersey light traps or Mosquito Magnets® (Woodstream Corp., Lititz, PA, USA) as described previously [23], and additional mosquito collections were conducted using the BG Sentinel Trap (BioQuip Products, Rancho Dominguez, CA, USA) or CDC light traps baited with dry ice used for the 2016 Zika virus survey. Mosquito species were separated based on morphological identification and pooled (1–35 specimens per pool) according to species, collection site and date. The specimens were stored at −80 °C or on dry ice during shipment.

### 2.2. Nucleic Acid Extraction, Random Amplification, Library Preparation and Sequencing

Mosquito pools were homogenized using glass beads and BioSpec Mini-BeadBeater 16 (Bio Spec Products Inc., Bartlesville, OK, USA). The mosquito homogenates were centrifuged and the supernatants were collected and treated with DNase I, Benzonase nuclease and RNase A [20]. Viral lysis and nucleic acid extraction were performed using QIAamp 96 Virus QIAcube HT kit (Qiagen Sciences, Germantown, MD, USA). 

RNA extracts were subjected to DNase I treatment, followed by reverse transcription polymerase chain reaction (RT-PCR) using anchored random octamer primers [24]. The PCR products were quantified by the Agilent 4200 TapeStation system (Santa Clara, CA, USA). Sequencing libraries were made using Nextera XT DNA library prep kits, using set A, B, C or D v2 indexes (Illumina, San Diego, CA, USA). Index sets were alternated between sequencing runs to avoid run-to-run carryover. Libraries were quantitated using TapeStation and pooled at equal molar concentrations. NGS was performed using an Illumina Miseq with MiSeq Reagent Kit v3 (600-cycle).

### 2.3. Metagenomic Data Analysis

MiSeq sequence read data were used in metagenomic analysis with an in-house de novo pipeline, as described previously [25]. The pipeline consisted of data pre-processing by quality filtering and adapter removal using cutadapt [26] and prinseq-lite [27]. De novo assemblies of reads were created using Ray Meta [28], followed by contig/scaffold assembly using Cap3 [29]. Contigs were classified using an iterative BLAST search against the NCBI nt database. In-house reference-mapping pipeline NGS_Mapper (https://github.com/VDBWRAIR/ngs_mapper) was then used to verify the existence of the viral contig sequences within pertaining sample reads. NGS_Mapper utilizes the BWA-MEM (https://arxiv.org/abs/1303.3997v2) assembler and several python scripts to map to a reference sequence and generate a FASTA consensus, run visuals, a VCF file, a BAM file and other statistical information. Geneious R10 (Biomatters Ltd, Auckland, New Zealand) and IGV [30] were used to visualize the output viral sequence and manually curate each nucleotide base position for false variant base calls and assembly errors.

Annotations of viral genome sequences were generated using both NCBI conserved domain searches integrated into Blastp functionality and InterPro, a protein sequence analysis and classification tool from EMBL-EBI [31]. Transmembrane regions were predicted using TMHMM v2.0 [32]. Endogenous viral elements (EVEs) were searched using a tBlastn against all cellular organisms with an E value maximum of 1e−10.

Phylogenetic analyses were done on the RNA-dependent RNA polymerase (RdRp) sequences for the novel viruses and related sequences in the GenBank database with >20% amino acid identity and >60% query sequence coverage. GenBank sequences with high overall distance to their respective dataset were excluded to allow for a more reliable phylogenetic analysis. Sequence alignment and phylogenetic tree building were done using MEGA 7 [33]. The amino acid sequences were aligned by MUSCLE. Models for evolutionary phylogenetic inference were picked based on the lowest corrected Akaike information criterion (AICc) scores for each respective alignment. The Le Gascuel model [34] with gamma distributed and invariant site rates (LG + G + I) was found to be the best fit for each dataset. Phylogenetic trees were computed using the Maximum Likelihood method with 500 bootstrap replicates.

### 2.4. Quantitative RT-PCR Assay

The Power SYBR Green RNA-to-Ct 1-Step Kit and the QuantStudio 7 Flex Real-Time PCR System (Applied Biosystems, Foster City, CA, USA) were used in the real-time RT-PCR assays. Primer set 1, YSLV1-2385F (5’ATGCGGAAAACCTATGGCCA3’) and YSLV1-2711R (5’TTGGGACCCAATTCTCGAGC3’), and primer set 2, YBV1-2844F (5’GCAACCCCAACATTGACCAC3’) and YBV1-3366R (5’TCCTCCTGCTGGGATAAGCT3’) (Integrated DNA Technologies Inc., Skokie, IL, USA) were designed based on RdRp gene sequences. The samples were either RNA extracts or 1:10 dilutions of the clear supernatants of mosquito homogenates. Infection rates were calculated by Maximum Likelihood Estimation (MLE), using PooledInfRate software [35] with 95% confidence and a scale of 1000.

### 2.5. Electron Microscopy

To prepare samples for imaging, the mosquito homogenate was centrifuged at 6000× *g* for 10 min and the supernatant was buffer exchanged and concentrated with phosphate-buffered saline (PBS) and Amicon Ultra Centrifugal Filters with a molecular cut-off (MWCO) of 100 K (MilliporeSigma, Burlington, MA, USA). Samples were stained using a 300 mesh Formvar Cu grid (Electron Microscopy Sciences Inc., Hatfield, PA, USA) and uranyl acetate, and examined with a JEM-1400 Transmission Electron Microscope (JEOL USA, Inc., Peabody, MA, USA). The images were recorded by an AMT camera (Advanced Microscopy Techniques Corp., Woburn, MA, USA).

## 3. Results

### 3.1. Identification of Known and Novel Viruses by Metagenomic Analysis

**Figure 1:**
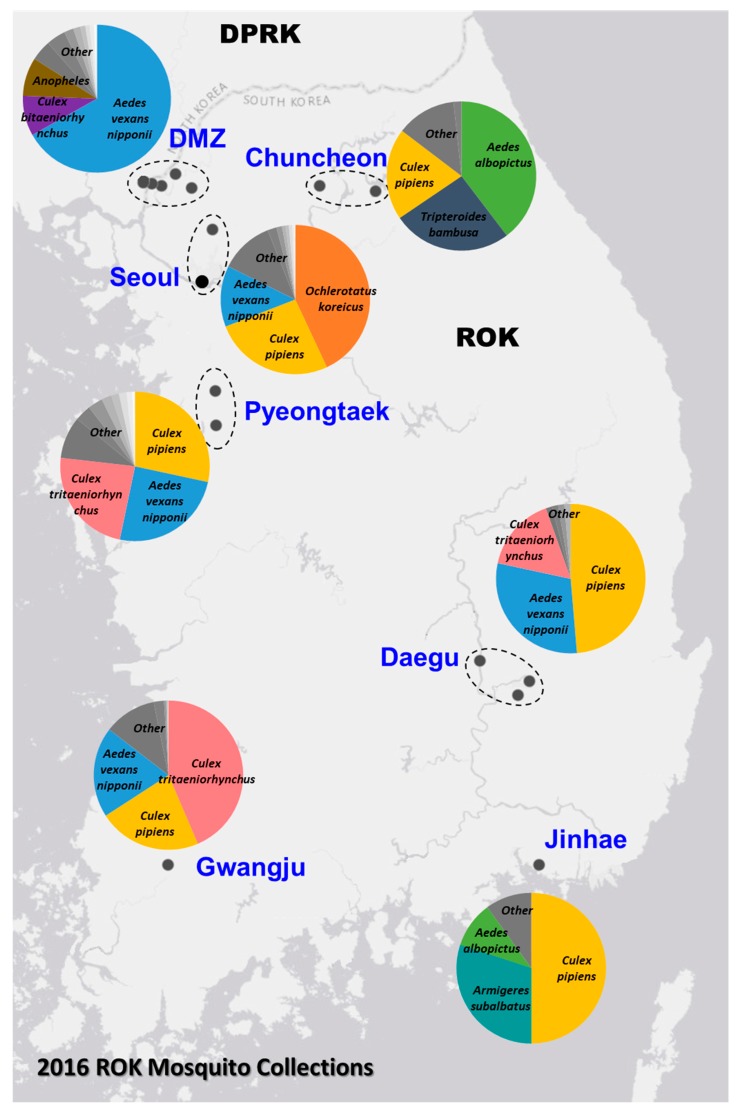
Mosquito collection from the Republic of Korea (ROK) in 2016. The top mosquito species collected by zone are depicted by the overlaid charts.

The Force Health Protection and Preventive Medicine, MEDDAC-Korea, conducted a comprehensive nationwide multi-location arthropod vector-borne disease surveillance program in the ROK. In 2016, a total of 4393 mosquito pools, containing 72,160 mosquitoes belonging to 24 species, were collected from 20 geographically diverse locations (Figure 1). The relative abundance of the trapped mosquito species varied among the sites, with *Ae. vexans nipponii, Culex pipiens* and *Cx. tritaeniorhynchus* being the most predominate species collected. The sampling sites represented diverse geographic areas of different population densities across the ROK, ranging from the densely populated urban areas (e.g., Yongsan U.S. Army Garrison (USAG) located in Seoul) to restricted rural regions near or in the demilitarized zone (DMZ).

In this study, unbiased sequencing was performed on 736 mosquito pools (6368 specimens) representing 16 species from 18 sites (Table S1). In total, NGS data of 412 million MiSeq reads were generated and applied to the metagenomic analysis to identify eukaryotic, bacterial and viral organisms present in the mosquito pools. From the 71 million non-eukaryotic reads identified, 19 million were from known viruses, seven million were from bacteria and 45 million were from unknown sources. Nucleotide sequence-based identification, using both the Blastn megablast and discontiguous megablast algorithms and the subsequent taxonomic annotations, resulted in the detection of a variety of viral sequences from many families, including 20 classified species belonging to the families *Alphaflexiviridae*, *Alphatetraviridae*, *Arenaviridae*, *Baculoviridae*, *Betaflexiviridae*, *Birnaviridae*, *Dicistroviridae*, *Flaviviridae*, *Iflaviridae*, *Mesoniviridae* (GenBank accessions MH520100–MH520106), *Nodaviridae*, *Partitiviridae*, *Parvoviridae*, *Reoviridae*, *Rhabdoviridae*, *Togaviridae*, *Tombusviridae*, *Totiviridae* and *Tymoviridae*, and new virus taxa which were reported as unclassified in GenBank. Furthermore, there were 3370 assembled sequence contigs with lengths of 500 nt or greater that did not have significant similarity found in the Blastn search. Further characterization of the sequences in the pooled samples is required to determine whether they contain novel viral genomes.

### 3.2. Discovery of Three Distinct Viruses in One Mosquito Pool, 16-0052

Pool 16-0052, which contained 16 *Ae. vexans nipponii* mosquitoes, was sequenced and a total of 2.5 million sequence reads and 508 million bases of data were obtained. Several large de novo sequence assembly contigs were found to have no significant similarity in the Blastn search. Using the blastx program, one contig was found sharing 33% amino acid identity with Hubei picorna-like virus 82 (KX883688), an unclassified RNA virus found in spiders collected in China in 2013 [13]. One contig was found sharing 83% and 58% amino acid sequence identities with two hypothetical proteins of the Wenzhou sobemo-like virus 4 (NC_033138.1), an unclassified RNA virus found in mosquitoes collected in China in 2013. Three of the unidentified contigs were found to share amino acid identities of 39%, 37% and 35% with three separate segments of Wuhan Mosquito Virus 2 (NC_031312.1), an unclassified RNA virus found in mosquitoes collected in China in 2012 [36].

Negative-staining electron microscopy of the concentrated homogenate supernatant showed the presence of particle sizes of approximately 100, 60 and 30 nM in pool 16-0052 (Figure S1). The approximate particle sizes of classified bunya-, picorna- and sobemoviruses are 100, 30 and 30 nm, respectively [37]. Faint surface spikes were visible around the 100-nm particle (Figure S1). However, due to the limited resolution in the captured image, characteristic virus structures were not clearly revealed. 

Based on the novelty of the sequences in pool 16-0052 and the identities of the deduced hypothetical proteins, three novel viruses were tentatively designated as Yongsan bunyavirus 1 (YBV1), Yongsan picorna-like virus 3 (YPLV3) and Yongsan sobemo-like virus 1 (YSLV1), according to the collection location and the tentative taxonomical classifications. The genome sequences were deposited in GenBank under accessions MH703045-MH703047, MH703048 and MH703049 respectively.

### 3.3. Genome Sequences and Phylogenetic Analysis of the Viruses

**Figure 2:**
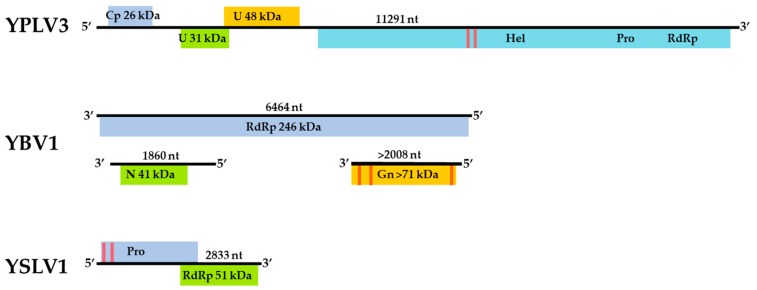
Genome maps of the three viruses, Yongsan bunyavirus 1 (YBV1), Yongsan picorna-like virus 3 (YPLV3) and Yongsan sobemo-like virus 1 (YSLV1). Colored boxes represent hypothetical protein reading frames. Proteins are labeled with putative functions based on conserved motifs and Blastp identities as undetermined (U); Viral coat protein (Cp); RNA Helicase (Hel); RNA-dependent RNA polymerase (RdRp); Nucleocapsid (N); Glycoprotein precursor (Gn); Protease (Pro). Red bars represent a high probability of transmembrane helices.

The assembled genome sequence of the YBV1 virus consists of three separate segments with lengths of 1860, 2008 and 6464 nt corresponding to the nucleocapsid, glycoprotein precursor and RNA-dependent RNA polymerase (RdRp) genes, respectively (Figure 2). The YBV1 nucleocapsid gene encodes for a 380-amino acid protein with 34% amino acid identity of with a viral nucleoprotein from Wuhan Mosquito Virus 2 (YP_009305134.1), which is the closest aligned sequence in the NCBI non-redundant (nr) protein database. The 638-amino acid partial protein encoded by the glycoprotein gene shares 37% amino acid identity with the closest aligned nr glycoprotein, also from Wuhan Mosquito Virus 2. One C-terminus transmembrane helix and two helices towards the N-terminus are predicted in the glycoprotein, consistent with known bunyaviruses and top NCBI aligned glycoproteins. Interestingly, the YBV1 glycoprotein coding sequence and the top six Blastp hits exhibited very similar lengths with an average of 699 amino acids, smaller than the typical bunyavirus M segment polyproteins. The more highly conserved 2133-amino acid YBV1 RdRp gene encoded protein shared 40% amino acid identity with the most closely aligned viral RdRp protein, which was from the Culex phasma-like virus. For all three YBV1 segments, most Blastp alignments largely consisted of insect viruses, with the top two being *Culex spp.* mosquito viruses, Culex phasma-like virus and Wuhan Mosquito Virus 2 (ASA47365.1, YP_009305135.1). To better elucidate the YBV1 host, a search for the endogenous viral elements (EVEs) against all cellular organisms was performed. The search yielded only flying insects, namely, *Bombus terrestris* (3.9e−11), *Bemisia tabaci* (5.4e−13) and *Rhagoletis zephyria* (1.4e−17).

The assembled YPLV3 genome sequence is 11.3-kb in length and encodes for three small hypothetical proteins and a polyprotein of 2401 amino acids (Figure 2). A protein motif search of the polyprotein determined the locations of genes for putative RNA helicase, protease and RdRp domains. ORF 1 was found to have a jelly-roll motif indicative of a viral coat protein but no sequences with significant similarity were found in Blastp searches. ORF 2 aligned exclusively to the hypothetical protein 2 of Hubei picorna-like virus 82 (HPLV82) (KX883688), a picorna-like virus found in spiders [13], with 24% amino acid identity. ORF 3 also matched with another protein from HPLV82, which has been previously reported as a possible capsid protein [33] with 30% amino acid identity. The polyprotein shares 33% amino acid identity with the aligned region of the closest Blastp result in the nr protein database, HPLV82, and 41% amino acid identity when searched with only the putative RdRp region. While atypical in length and with an ORF layout similar to picornavirus (7–9 kb and a single ORF), YPLV3 shares similar characteristics with the newly proposed family *polycipiviridae* (polycistronic picorna-like viruses). Despite a very divergent sequence identity, YPLV3 shares a similar genome length, multiple short ORFs and polypeptide motif orientation with members of *polycipiviridae* [38]. A search for EVEs resulted in no hits that met the E value maximum of 1e−10. 

The 2833-nt YSLV1 has a RdRp-encoding reading frame which shares 83% amino acid identity with the closest Blastp hit, mosquito virus Wenzhou sobemo-like virus 4 (NC_033138.1) [13], while the other ORF shares 54% amino acid identity with the same Blastp aligned organism. YSLV1 was found to have the characteristic overlapping reading frame, −1 frameshift and protein layout of known sobemoviruses, but it is missing two small N- and C-terminus reading frames typical of known plant strains [37]. Examination of the top two blast aligned organisms (Wenzhou sobemo-like virus 4 and Hubei mosquito virus 2), both found in mosquito hosts, also showed this −1 frameshift and lack of N- and C-terminus reading frames. Many invertebrate-associated sobemo-like viruses, but no known plant sobemovirus genomes, were aligned in the top 100 blast hits. No possible EVE matches were observed.

**Figure 3:**
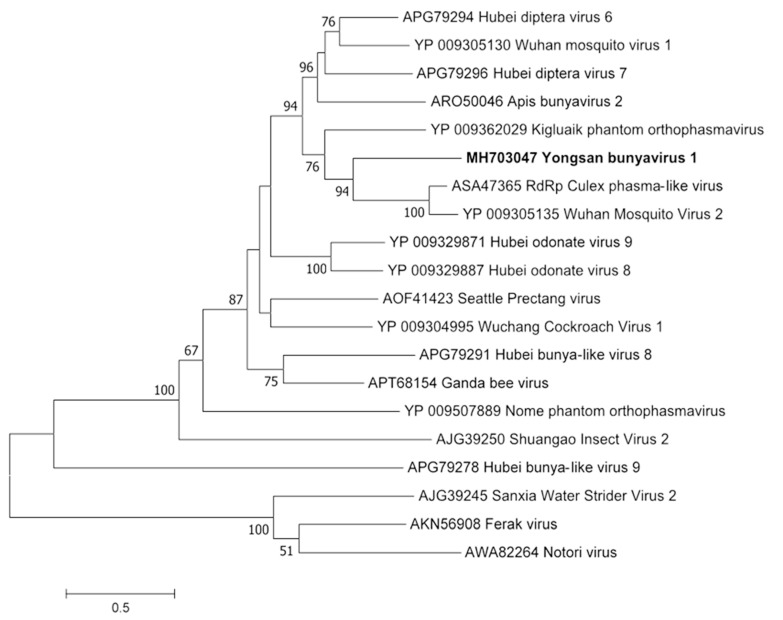
The phylogenetic tree of Yongsan bunyavirus 1 and related viruses. The analysis was inferred by using the Maximum Likelihood method based on the Le Gascuel 2008 model. Branch bootstrap values above 50 are shown and were based on 500 replicates. The tree is drawn to scale, with branch lengths measured in the number of substitutions per site. The tree is midpoint rooted.

**Figure 4:**
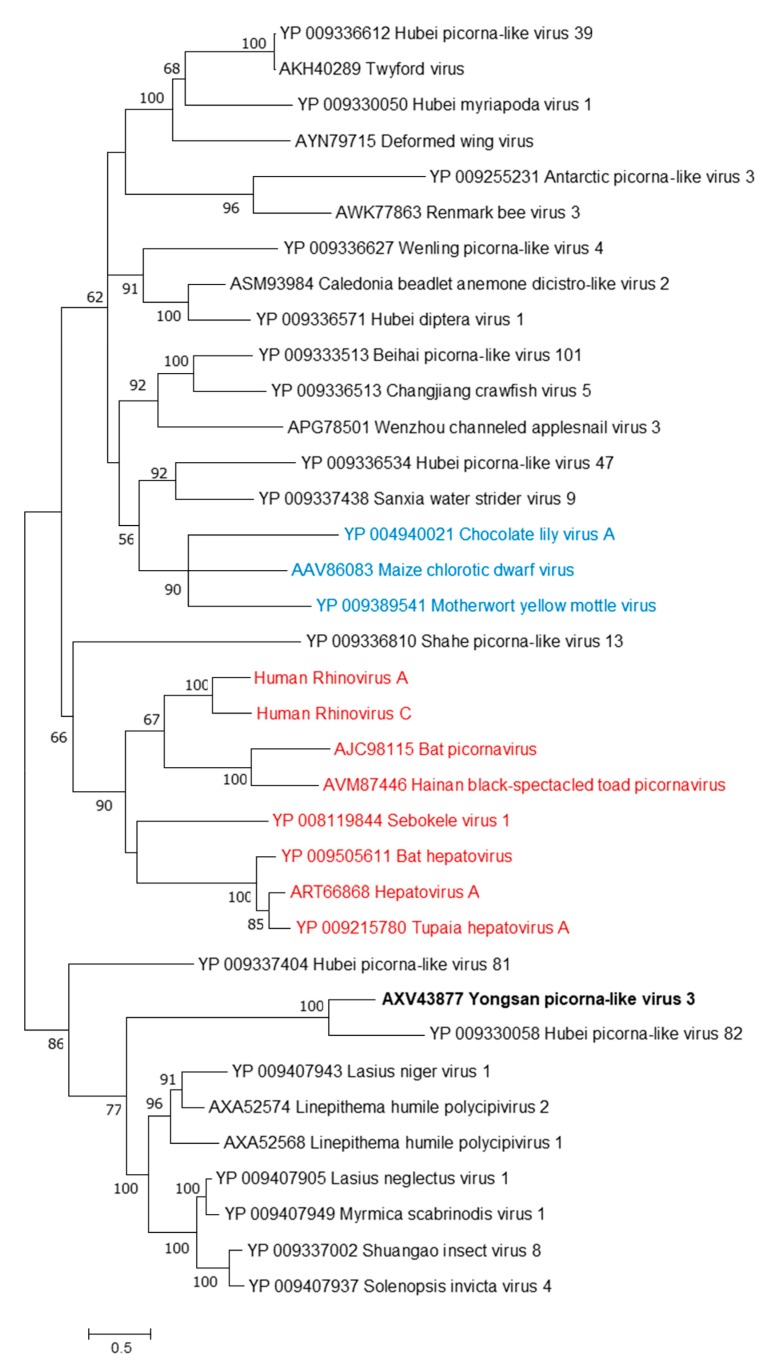
The phylogenetic tree of Yongsan picorna-like virus 3 and related viruses. The analysis was inferred by using the Maximum Likelihood method based on the Le Gascuel 2008 model. Branch bootstrap values above 50 are shown and were based on 500 replicates. The tree is drawn to scale, with branch lengths measured in the number of substitutions per site. The tree is midpoint rooted. Viruses are colored based on host: invertebrate (black), vertebrate (red) and plant (blue).

**Figure 5:**
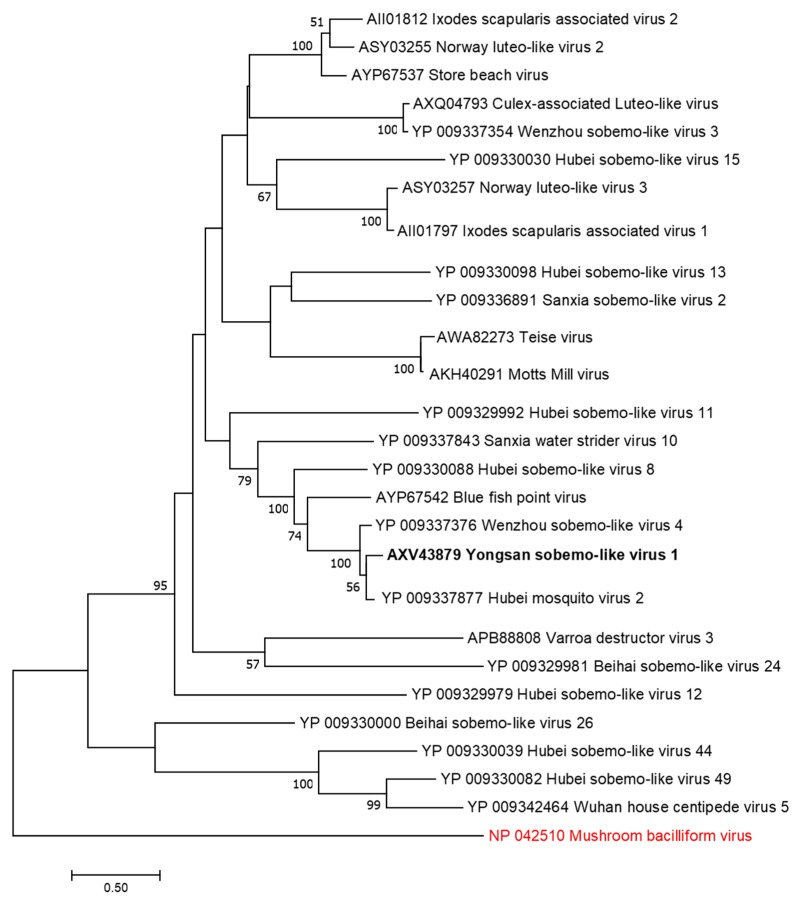
The phylogenetic tree of Yongsan sobema-like virus 1 and related viruses. The analysis was inferred by using the Maximum Likelihood method based on the Le Gascuel 2008 model. Branch bootstrap values above 50 are shown and were based on 500 replicates. The tree is drawn to scale, with branch lengths measured in the number of substitutions per site. The tree is midpoint rooted. Viruses are colored based on host: invertebrate (black) and fungi (red).

Phylogenetic analyses were done on the RdRp region of all three viruses. Non-redundant sequences that shared over 20% amino acid identity and over 60% query coverage were included in these analyses (Figure 3, Figure 4 and Figure 5). Unsurprisingly, the vast majority of similar sequences came from genomes associated with invertebrate hosts and vectors. Sequences that met the inclusion criteria for YBV1 were exclusively viruses associated with flying insects (Figure 3). Of the three viruses, YPLV3 was the only virus shown to be phylogenetically related to vertebrate viruses, including human rhinoviruses and a mammalian hepatovirus (Figure 4). YSLV1 matched with 84 genomes associated with invertebrate hosts (1 fungi), and, of those, almost all were insect- and arthropod-associated viruses (Figure 5). Due to the novel and diverse nature of these viruses, many were extremely distant from all others and failed to form reliable clades in the analysis. Due to this, some were eliminated to reduce redundancy and increase overall tree reliability. 

### 3.4. Wide Distribution of the Viruses

To better understand the diversity and epidemiology of these viruses, sequence data from all 736 pools were screened for the presence of YBV1, YPLV3 and YSLV1 sequences (Table 1). The in silico screening of sequenced pools was performed by local Blastn searching against a database made from the assembled contigs from all 736 sample pools. To minimize the possibility of false positives through low levels of cross-contamination or systematic sequencing error such as index hopping, no pools with fewer than 100 viral reads were considered positive.

YBV1 sequences were found in 19 out of 70 (27.1%) sequenced *Ae. vexans nipponii* pools and only 4 out of 666 (0.6%) other mosquito pools. Of the 23 YBV1 positive pools, sequence data for 20 pools contained all L, M and S segments, while three pools did not contain any reads for the M segment. The average YBV1 read counts for the 20 pools containing three segments are 24,059, 1424 and 3047 for the L, M and S segments respectively. Of the three pools without M segment reads, the singular *Ae. vexans nipponii* pool had low L/S read counts of 1885/107. The other M segment lacking *Cx. vagans* and *Cx. tritaeniorhynchus* pools had L/S read counts of 21,093/ 22,398 and 6509/176. Most identified pools (22/24) shared >98% nucleotide identity with the 16-0052 strain for all segments. 

YPLV3 sequences were found in three mosquito pools that were all collected from Yongsan USAG. A total of 177,229 YPLV3 reads were identified in pool 16-0052. The additional *Ae. vexans nipponii* pool 16-0059, which shares 99% nucleotide identity to 16-0052, was found to contain 130,542 YPLV3 reads. The additional *Cx. pipiens* YPLV3 read-positive pool, 16-0123, provided only 1056 YPLV3 reads, which was only enough for partial coverage. 

A total of 23/70 (32.9%) *Ae. vexans nipponii* pools along with one *Ochlerotatus koreicus* pool and four *Culex* spp. pools were found to have YSLV1 sequence reads. The mean YSLV1 read count per positive pool was 38,627. Due to the small genome size and high read yield, the depth of coverage in the YSLV1 read-positive pools was sufficient to assemble 25 near complete genomes. YSLV1 viruses of pools from different mosquito species and different localities shared nucleotide identity greater than 98%. 

The metagenomic NGS data suggest that YBV1 and YSLV1 have a high prevalence in *Ae. vexans nipponii*. The RdRp primers were designed and used for RT-PCR assays of 52 sequenced and 205 additional *Ae. vexans nipponii* pools (3493 total mosquitoes) (Table S1). From the 257 RT-PCR-tested pools, there were 167 (65%) positive for YBV1 with Ct values ranging from 22.6 to 37.5, 159 (62%) positive for YSLV1 with Ct values ranging from 16.7 to 37.3 and 121 (47%) positive for both. Melting curve analysis showed melting point temperatures around 77 °C (76.6–77.3 °C) and 79 °C (79.1–80.3 °C), respectively, for YBV1 and YSLV1 amplicons (Figures S2 and S3). Regression analysis showed a statistically significant correlation between increasing pool size and the pool infection rate with *p*-values of 0.0004 and 0.0001 for YBV1 and YSLV1, respectively. The infection rates calculated by MLE, a statistical analysis used to determine the most likely rate (infection probability per mosquito) by taking into account pool sizes, were found to be 8.8% for YBV1 and 8.0% for YSLV1 (Table 2). YBV1 and YSLV1 RNA-positive pools by collection zones are also summarized in Table 2.

## 4. Discussion

With worldwide travel, global trade, urbanization, agriculture development and environmental changes, both stationary and naïve populations are at increased risk for emerging and reemerging diseases [39,40]. Arboviruses have been responsible for 30% of emerging diseases in recent years [41], making it important to broaden the surveillance of mosquitoes and other biting arthropods from the detection of a limited number of specific pathogens to a more comprehensive and discovery-orientated approach. In this study, the sites of mosquito collections ranged from dense population centers to rural areas and represent all geographic regions in the ROK, allowing for a diverse array of sampling environments and enabling more complete surveillance. Aside from a health protection perspective, these analyses are important for a better understanding of viral diversity and the ecological role viruses play in their environment.

Unbiased metagenome sequencing has become an important tool in virus discovery and surveillance. Metagenomic analysis is independent of viral culture and isolation and does not rely on previous knowledge of the viruses. In this study, three near-complete novel viral genomes were found in one pool of 16 *Ae. vexan nipponii* mosquitoes and, with subsequent screening, confirmed a high abundance of two of these viruses throughout geographically diverse collection sites in the ROK. The sequence alignments, structural characteristics and putative protein motifs were used to associate these three unclassified viruses to the viral family *Bunyaviridae*, order *Picornavirales* and genus *Sobemovirus* (family unassigned), albeit with highly divergent sequence and structural characteristics. 

*Ae. vexans nipponii* is a known carrier of Chaoyang flavivirus, tick-borne encephalitis virus and several parasites, e.g., *Dirofilaria repens* and *D. immitis* [42,43,44]. *Aedes vexans nipponii* is a potential vector for known and unknown human pathogens due to its zoophilic nature and willingness to bite humans, emphasizing the importance of understanding its virome. The family *Bunyaviridae* represents an expansive and diverse family of negative-sense viruses mostly comprised of three single-stranded RNA (ssRNA) segments [45]. Members of the family *Bunyaviridae* encompass many important and recent emerging human pathogens, e.g., Hantaviruses, tick-transmitted Crimean-Congo Hemorrhagic fever and mosquito-transmitted La Crosse encephalitis virus, and are also represented by a high abundance and diversity of arthropod-specific viruses [2,13,46,47,48,49]. The family *Picornaviridae* is a large and diverse family comprised of positive-sense ssRNA viruses—many of which infect humans and a wide array of animals [45]. Recent metagenomics work has found a great variety of novel picorna-like viruses in invertebrates including some found in mosquitoes [13,50]. The genus *Sobemovirus* encompasses a relatively small set of positive-sense ssRNA viruses, which are exclusively plant viruses [51]. Insects are known vectors of sobemoviruses through mechanical transmission and wounding of plants [51]. There is a wealth of recently proposed sobemo-like viruses from the metagenomic analyses of invertebrates and a sobemo-like virus has recently been suggested as a bat virus [13,47,52,53]. 

The host specificities of these viruses were not determined in this study. Instead, predictions were made based on endogenous viral element (EVE) analysis along with other observations. For YBV1, not only is *Bunyaviridae* a well-established insect and mosquito virus family [37], EVE hits to other flying insects help infer common host viral ancestry. EVE matches in insects combined with the high abundance of YBV1 found in the tested mosquitoes suggest a virus that is able to replicate in mosquito hosts. Even though no EVEs were found for YPLV3 and YSLV1, all three viruses showed clear association with other invertebrate-associated (mostly unclassified) viruses. YPLV3 was the only one of the three viruses that aligned with vertebrate viruses, some of which included human rhinoviruses (A, B and C) and hepatovirus A, but these were more distant when compared to aligned invertebrate viruses. In addition to the three YPLV3 viruses identified in *Aedes* and *Culex* mosquitoes from Yongsan USAG, several additional picorna-like viruses were found in the ROK mosquitoes by our group (MH703060, MH703059, MH703052, MH703051, MH703050). Together with other reports, a large and increasing number of picorna-like viruses have been identified from mosquitoes and other arthropods, further suggesting that YPLV3 is a mosquito virus. Without clear evidence of arthropod-infecting-classified sobemoviruses, host elucidation for YSLV1 is more speculative. Arthropods have been observed as vectors for plant sobemoviruses. YSLV1 has sequence similarity with >100 exclusively invertebrate-, mainly arthropod-, associated unclassified sobemo-like viruses. None of these viruses were found in plants or demonstrated to be infectious to plants, raising the possible need for additional arthropod-specific viral taxa classifications within the family *Solemoviridae.* More studies, including viral isolation and infectivity assays in cell and tissue, are required to determine the host specificity of these viruses as well as querying for the remote possibility of vertebrate pathogenicity.

The widespread nature of YBV1 and YSLV1 in *Ae. vexans nipponii* illustrates the ability of certain viruses to spread and persist among mosquito species. Nucleic acids for both viruses were found at all collection zones and throughout the collection timeframe (May–October). A quick screening of metagenomics data from 2008, 2012 and 2017 ROK mosquito pools also showed an abundance of YBV1 and YSLV1 sequences in *Ae. vexans nipponii* during those years (data not shown). Although these are very taxonomically different viruses, the prevalence was similarly high with MLE infection rates of 8.8% and 8.0% for YBV1 and YSV1 respectively. While the high infection rates shown by the qRT-PCR and NGS data are corroborative, additional confirmatory tests—e.g., Sanger sequencing or Taqman-based assays—can be used to confirm the specificity of the qRT-PCR assay and the prevalence rates described herein. Due to the high infection rate, pools having both viruses were common and a significant positive correlation between the pool size and infected pools is observed, as expected. Future work to determine interactions between the two and other viruses would have to focus on a single mosquito specimen.

These data, in addition to other studies [13,36,47,48,52,54], highlight the richness of the insect virome and the insufficiency of virus taxonomy. The new challenge of classifying the large and increasing number of arboviruses in a timely and systematic manner has yet to be fully addressed. The focus of past studies has mostly been devoted to the characterization of isolated viruses and it has not been until recent advances in sequencing technology that these viromes are being explored at a large scale [55]. The majority of viruses found similar to the ones discovered herein were discovered after 2013. Notably, a recent study by Shi, M et al. [13] found 1445 novel viruses in 220 invertebrate species, which were heavily represented in our phylogenetic analyses and served to fill many gaps. Recent studies focusing specifically on mosquitoes have found a great deal of unclassified viral diversity among the family.

The viruses described in this paper are highly abundant, yet they can only be classified tentatively to known families. The sequence data associate them almost exclusively with other recently discovered and unclassified viruses. YBV1 and its top NCBI matches, which fit more neatly into a known family compared to YPLV3 and YSLV1, are extremely divergent from classified bunyaviruses. It is only with the very recent proposal of the *Polycipiviridae* family in the order *Picornavirales* that YPLV3 and several other insect-associated picorna-like viruses can be putatively assigned to a family, and even within these proposed polycipiviruses, there are significant structural and sequence differences [33]. Moreover, classified sobemoviruses consist of a relatively small number of plant viruses, yet the data herein and in other recent studies show that there is abundant sobemo-like diversity. 

Viral genetic exchange is commonly observed in invertebrate pathogens [13]. The widespread and abundant nature of pathogenic and nonpathogenic agents as well as their potential vertical transmission suggest a high level of viral coinfection and a chance of recombination events. It is rational to speculate that the enormous virome diversity observed in mosquitoes and other arthropod vectors make bloodsucking insects a substantial source of emerging viruses. The vast majority of the viruses identified recently in metagenomic analysis were not characterized and the host susceptibility and the relevance of these viruses to human or animal diseases are largely unknown. There is a possibility that some of these unknown viruses have acquired infectivity to vertebrates and are potential pathogens. Further study, including host specificity tests in cells and animals and serological assays of human or animal bloods, will shed light on whether these arboviruses pose a real threat to public health.

Nevertheless, viruses, though nonpathogenic by themselves, may alter infection, transmission and pathogenicity of vector-borne pathogens. Viral coinfection and virus–virus interactions can have significant effects on virus–host dynamics, transmissibility and the infectivity of viruses [56]. Pathogenic effects on the host vector can also contribute to the ability of that vector to transmit other diseases. *Culex* flavivirus (CxFV), a *Culex*-specific virus, has been reported to decrease the replication of West Nile virus (WNV) in CxFV-infected mosquitoes [57]. The insect-specific Palm Creek virus has been shown to reduce WNV and Murray Valley encephalitis titers in an *Aedes* cell line C6/36 by 10–43-fold [58]. Some viruses have been shown to have no effect on the transmissibility of coinfected viruses (e.g., Zika and chikungunya have a negligible effect on the transmissibility of each other), while other reports have shown a positive increase in infection between some viruses in coinfected mosquitoes [59,60]. From a disease prevention standpoint, vertically transmitted viral or non-viral pathogens in mosquitoes could be important for future disease prevention efforts. *Wolbachia* spp., a prevalent vertically transmitted insect bacterial pathogen, reduces the ability of several flaviviruses to infect mosquitoes and the release of *Wolbachia*-infected mosquitoes has been used to suppress endemic dengue transmission [61,62]. 

With much still unknown about the mosquito virome, even less is known about potentially health-relevant interactions of the diverse and abundant viruses that comprise it. It would be beneficial to further test the virus–virus and host interactions that these and other highly abundant unclassified viruses have on known human pathogens and their vectors. The high possibility of vertical transmission and the extreme abundance of YBV1 and YSLV1 make them good candidates for further study.

This study showed the discovery of three significantly novel viruses with two being highly prevalent in *Ae. vexans nipponii*. Our ongoing and future work in this metagenomics study will likely lead to the discovery of many more arboviruses. The data from this and other related studies will ultimately enable the creation of a map featuring full mosquito viromes closely cross-referenced to the demography of all mosquito species in the ROK. Considering the rapid progress in the field of metagenomics, it is feasible to integrate similar information from all regions, countries and continents to build a global database in the near future. Such a comprehensive and dynamic database, together with robust data mining and modeling tools, holds great promise in the meaningful simulation of arbovirus migration and possibly an operational projection of endemic areas and forthcoming transmission hot spots for infectious diseases caused by arbovirus pathogens.

## Figures and Tables

**Table 1:** Mosquitoes analyzed using metagenomic sequencing and the number of positive sequenced pools found for Yongsan picorna-like virus 3 (YPLV3), Yongsan bunyavirus 1 (YBV1) and Yongsan sobemo-like virus (YSLV1) reads.

			Number of Positive Pools			
Species	Number of Mosquitoes in the Pools	Number of Pools Sequenced	YPLV3	YBV1	YSLV1	Total
*Culex pipiens*	1942	120	1	1	2	2
*Culex tritaeniorhynchus*	1349	101		1		1
*Aedes vexans nipponii*	1096	70	2	19	23	32
*Aedes albopictus*	553	80				
*Mansonia uniformis*	498	76				
*Coquillettidia ochracea*	228	45				
*Ochlerotatus koreicus*	221	67		1	1	2
*Culex inatomii*	123	46			1	1
*Culex orientalis*	105	35			1	1
*Culex vagans*	101	14		1		1
*Culex bitaeniorhynchus*	88	57				
*Culiseta nipponica*	42	8				
*Armigeres subalbatus*	17	12				
*Ochlerotatus dorsalis*	3	3				
*Aedes lineatopennis*	1	1				
*Culex sasai*	1	1				
Total	6368	736	3	23	28	40

**Table 2:** RT-PCR-positive *Aedes vexans nipponii* pools by collection zone. Infection rates were calculated by Maximum Likelihood Estimation (MLE) using PooledInfRate (Biggerstaff, CDC, *www.cdc.gov/westnile/resourcepages/mosqSurvSoft.html*).

Sample Location	No. of Mosquitoes	No. of Pools	YBV1		YSLV1	
			Positive Pools	Infection Rate (%)	Positive Pools	Infection Rate (%)
Daegu	22	14	3	13.6	8	40.3
Pyeongtaek	1740	118	81	8.7	72	6.9
Gwangju	332	36	22	10.0	16	5.9
Seoul	1399	89	61	8.1	63	9.6
**Total**	**3493**	**257**	**167**	**8.8**	**159**	**8.0**

## Supplementary Materials

The following are available online at https://www.mdpi.com/1999-4915/11/3/222/s1, Table S1: List of mosquitoes analyzed using metagenomics sequencing and/or RT-PCR. Figure S1: Negative-stained electron microscopy image of ultrafiltration concentrated homogenate supernatant from mosquito pool 16-0052. Figure S2: Melting curve analysis of YBV1 qRT-PCR amplicons. Figure S3: Melting curve analysis of YSLV1 qRT-PCR amplicons.
