# Supplementary material for: Metagenomic Analysis Reveals Three Novel and Prevalent Mosquito Viruses from a Single Pool of Aedes vexans nipponii Collected in the Republic of Korea
Source: Viruses. 2019 Mar 5;11(3):222. doi: 10.3390/v11030222 (PMC6466275; doi:10.3390/v11030222)
Supplement: Supplementary file 1 [file viruses-11-00222-s001.zip › Supplemental/viruses-430597-Figures S1-S3.pdf]

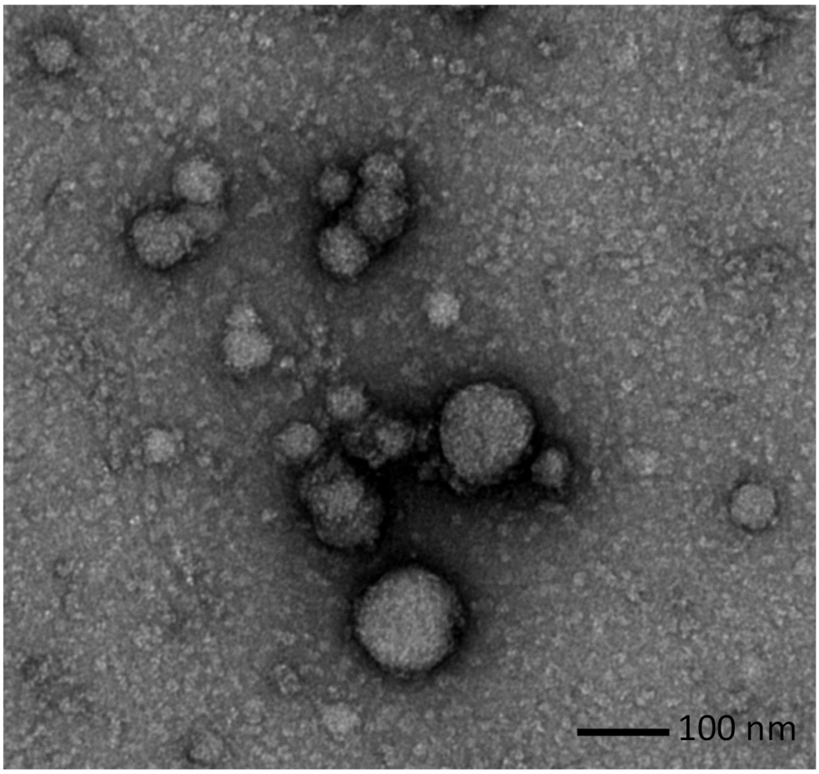

Figure S1. Negative stained electron microscopy image of ultrafiltraion concentrated homogenate supernatant from mosquito pool 16-0052. A scale of 100 nm is indicated by the black bar.

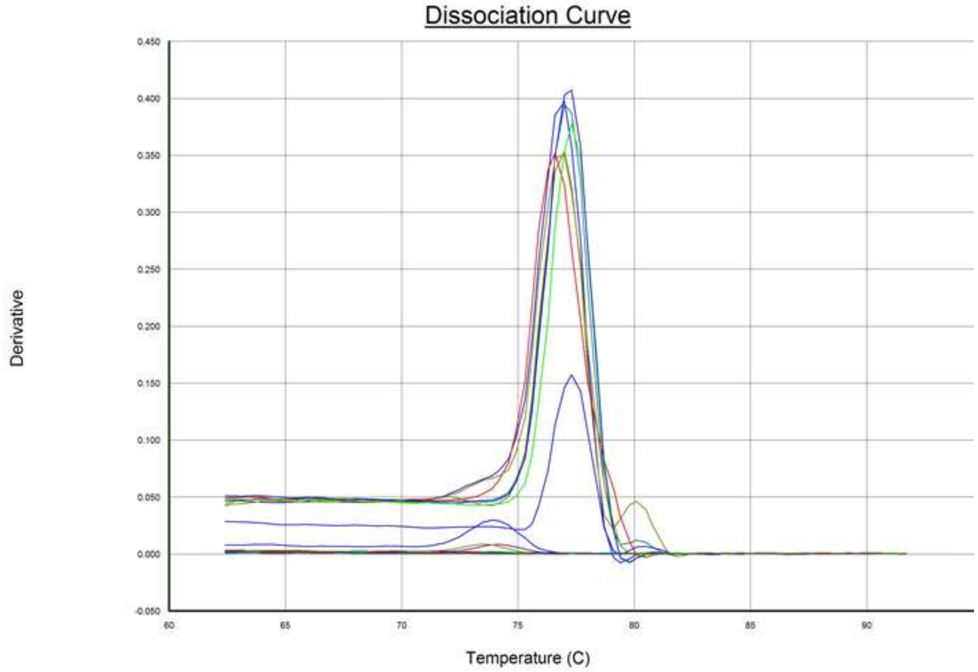

Figure S2. Melting curve analysis of YBV1 qRT-PCR amplicons from 20 total reactions (8 positive/12 negative). Positive samples are represented by a melting point temperature of around 77 °C and negative samples are represented by mostly flat lines.

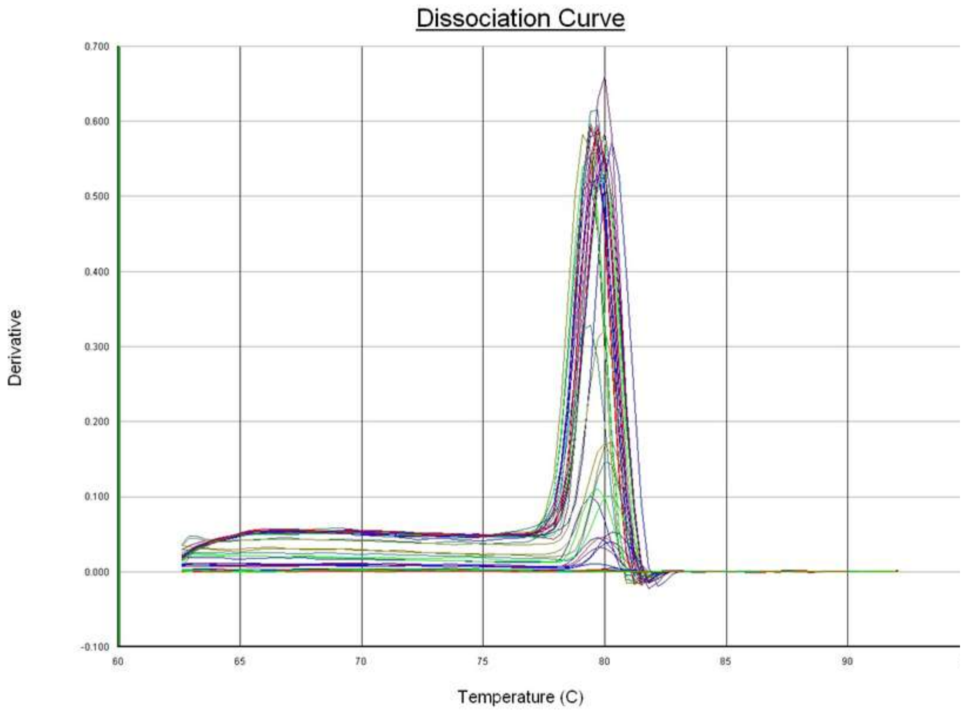

Figure S3. Melting curve analysis of YSLV1 qRT-PCR amplicons from 92 total reactions (38 positive/54 negative). Positive samples are represented by a melting point temperature of around 79 °C and negative samples are represented by mostly flat lines.
